# Chondrosarcome secondaire à localisation fibulaire rare: à propos d'une observation

**DOI:** 10.11604/pamj.2015.22.159.7992

**Published:** 2015-10-20

**Authors:** Soufiane Guelzim, Ahmed El Bardouni

**Affiliations:** 1 Service de Chirurgie Orthopédique et Traumatologie, CHU Ibn Sina, Rabat, Maroc

**Keywords:** Chondrosarcome, fibulaire, histogénèse cartilagineuse, Chondrosarcoma, fibular, cartilage histogenesis

## Image en medicine

Le chondrosarcome regroupe plusieurs formes anatomocliniques de tumeurs à histogénèse cartilagineuse. Il s'agit d'une tumeur maligne dont les cellules tumorales sont associées à une matrice cartilagineuse. Le chondrosarcome est souvent primitif mais dans 10% des cas, il peut survenir sur des tumeurs bénignes préexistantes, essentiellement les exostoses ostéogéniques et les chondromes. Le risque de transformation maligne est très faible pour une exostose solitaire. Dans la maladie des exostoses multiples, le risque est de l'ordre de 15%. Les auteurs rapportent un cas de chondrosarcome secondaire du péroné distal chez un jeune patient de 19 ans, dans les suites d'une dégénérescence d'une exostose solitaire de l'extrémité inférieure du péroné droit évoluant depuis 9 mois. Devant l'augmentation du volume de l'exostose devenue douloureuse (A), la transformation maligne a été de suite suspectée et le patient a bénéficié d'un bilan d'extension osseux (B), extra-osseux et d'une biopsie ayant objectivé un chondrosarcome de grade 1. Le patient a bénéficié d'une résection chirurgicale large (C,D), selon les règles habituelles de la chirurgie carcinologique avec arthrodèse tibio-astragalienne (E) pour pallier à l'instabilité latérale de la cheville occasionnée par cette résection. Les suites opératoires ont été simples (F).

**Figure 1:**
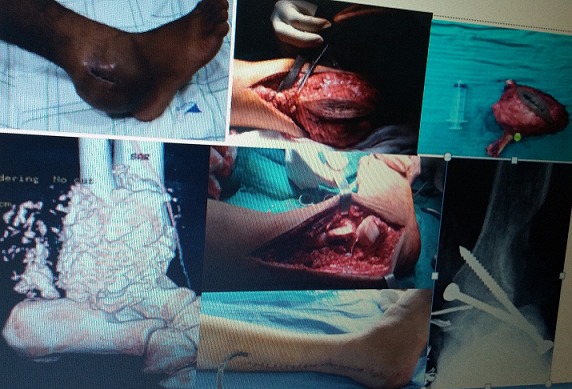
A): Image clinique de la cheville droite; B): TDM avec reconstruction de la cheville droite; c): Image peropératoire après résection large; D): pièce opératoire; E): Résection large avec arthrodèse tibio-astragalienne; F): Image clinique post opératoire

